# Cognitive Functions in Schizophrenic Patients: A Case-Control Tunisian Study

**DOI:** 10.1192/j.eurpsy.2025.2267

**Published:** 2025-08-26

**Authors:** N. Smaoui, D. Jardak, R. Feki, S. Omri, I. Gassara, M. Bou Ali Maalej, N. Charfi, J. Ben Thabet, M. Maalej, L. Zouari, L. Triki

**Affiliations:** 1 psychiarty C, Hedi Chaker University Hospital; 2 Functional Explorations, Habib Bourguiba University Hospital SFAX, Sfax, Tunisia

## Abstract

**Introduction:**

Cognitive impairments in schizophrenic patients are present from the first psychotic episode and remain relatively stable over time. These cognitive impairments primarily affect memory, attention, executive functions, and social cognition.

**Objectives:**

The aim of this study was to assess cognitive functions in schizophrenic patients by comparing them to healthy controls.

**Methods:**

Methods: We conducted a cross-sectional, descriptive, and analytical case-control study. It included 15 schizophrenic patients and 15 healthy controls. The study was carried out at the Psychiatry « c » Department outpatient unit at Hedi Chaker University Hospital in Sfax, Tunisia. Both cases and controls underwent interviews to answer predefined questionnaires. We used the Screen For Cognitive Impairment in Psychiatry (SCIP) scale in its literary Arabic version for the assessment of cognitive functions.

**Results:**

The average scores for the total SCIP (ST) and its five subscales (Verbal Learning Test-Immediate (VLT-I), working memory (WMT), verbal fluency (VFT), verbal learning-Test-delayed (VLT-D), and processing speed Test (PST)) were 37.40, 12.87, 14.27, 3.93, 2.47, and 3.93, respectively, for the cases, and 47.27, 15, 18.13, 5.40, 4.33, and 4.40, respectively, for the controls. The cases had significantly lower total SCIP scores than the controls (p=0.05), specifically in the WMT (p=0.02) and VLT-D (p=0.01) subscales. There was no significant difference between the two groups in the VLT-I (p=0.241), VFT (p= 0.202), and PST (p=0.598) subscales.

**Conclusions:**

This study found that cognitive deficits in schizophrenic patients primarily involved impairments in working memory and verbal learning-delayed recall. Early screening for cognitive impairments in these patients should be systematic to specify the deficits and to hasten the integration in the neurocognitive training programs.

**Disclosure of Interest:**

None Declared

